# The rhythm of effective entrepreneurs’ decision-making process. The pathways of alertness scanning and search and cognitive style. A mediation model

**DOI:** 10.1007/s11365-021-00759-1

**Published:** 2021-08-16

**Authors:** Sara Sassetti, Vincenzo Cavaliere, Sara Lombardi

**Affiliations:** 1 Department of Political Science, University of Pisa, Via Filippo Serafini, 3, 56126 Pisa, Italy; 2 Department of Economics and Management, University of Florence, Building D6, Via delle Pandette 9, 50127 Florence, Italy

**Keywords:** Entrepreneurial alertness, Rational style, Intuitive style, Decision-making effectiveness

## Abstract

How can entrepreneurs be effective when making decisions? To enrich current research on entrepreneurship and cognition, the present study shows how alertness and decision making are closely related. Prompted by the scant attention that scholars have paid to the link between alertness and the pathways of entrepreneurs’ thought, it proposes that being alert by adequately scanning and searching for information is likely to increase decision-making effectiveness. Distinguishing between rational and intuitive cognitive styles and based on a sample of 98 Italian entrepreneurs from small and medium manufacturing companies, the analysis shows that while a rational cognitive style significantly mediated the relationship, intuition did not play a role in shaping entrepreneurial decision-making effectiveness. The results suggest that developing individual alertness might not be sufficient for entrepreneurs to make effective decisions; a rational cognitive style might also be a key mechanism shaping this association.

## Introduction

The emergence of new ideas and how they can lead to commercializable opportunities are central to the field of entrepreneurship (Baron, 2006; Shane & Venkataraman, 2000; Short et al., 2010). Explanations of how new opportunities emerge include information, prior experiences, personal disposition, and the acquisition of specific information (Gaglio & Katz, 2001; Shane, 2000; Shepherd et al., 2007). Furthermore, the discovery of new opportunities has been linked to personal alertness, that is, the capacity to be aware of the signals and information that derive from certain contexts (Tang et al., 2012).

Entrepreneurial alertness is composed of three dimensions: scanning and searching, association and connection, and evaluation and judgment (Tang et al., 2012). Alert scanning and searching involves constantly scanning the environment and searching for new information, changes, and shifts that have been overlooked by others. The second dimension, alert association and connection, involves pulling together disparate pieces of information and building them into coherent alternatives. The third dimension involves making evaluations and judgments about the new changes, shifts, or information and deciding if they might lead to a business opportunity with profit potential. Of these alertness dimensions, the scanning and search component is fundamental because it is conducted at each stage of the opportunity recognition, development, evaluation, and exploitation process (Tang, 2016). Indeed, screening and searching for new information help decision makers to evaluate and make decisions based on different alternatives, so that is more probable that the best of the options will be chosen (Beach, 1990; Tang, 2016) and that more effective choices will be made. Scanning and searching has been seen as the main component that allows entrepreneurs to organize and interpret information in various domain of knowledge (Gaglio & Katz, 2001), developing cognitive frameworks to build the knowledge structure that lies at the base of entrepreneurial decisions (Mitchell et al., 2007; Tang, 2016). Knowledge structures are one of the main building blocks of the definition of entrepreneurial cognition; they are used “to make assessments, judgments, or decisions involving opportunity evaluation, venture creation, and growth” (Mitchell et al., 2002, p. 97). This definition highlights a further important element in the literature on entrepreneurial cognition: decision making. Kirzner (1979, 1985, 1992), the father of the concept of entrepreneurial alertness, consistently explained that alert and non-alert entrepreneurs can be distinguished by the way they make decisions about their current circumstances. Therefore, alertness and decision-making are closely related (Roundy et al., 2018). Nevertheless there is a paucity of studies of the effect of alertness on the decision-making process (Chavoushi et al., 2020). Most research has focused on the strong link that exists between alertness and entrepreneurial success (Amato et al., 2017; Baron & Markman, 2003). It has been shown that individuals high in entrepreneurial alertness are more likely to form an intention to start a business (Van Gelderen et al., 2008) and are more likely than individuals low in alertness to act on such intention (Shane et al., 1991). Furthermore, empirical evidence suggests that alertness is positively related to a firm’s innovativeness (Tang et al., 2012). In this context, it is important to note that identifying and selecting the most appropriate and potentially profitable opportunities for a new business are among the most important activities carried out by entrepreneurs (Stevenson et al., 1985). However, little attention has been paid to the link between alertness and the pathways of entrepreneurs’ thought, namely decision making, and the effectiveness of decisions that allow entrepreneurs to be successful (Zhao et al., 2020).

Moreover, scholars agree that entrepreneurial alertness is a cognitive capability possessed by entrepreneurs that enables them to notice venture opportunities (Gaglio & Katz, 2001). Great efforts have been made to incorporate cognitive theories into entrepreneurial alertness studies, such as pattern theory (Baron, 2006), schema theory, and attention theory (Valliere, 2013). So far, however, little heed has been paid to the process that relates entrepreneurial alertness to the pathways of thought and the associations between different cognitive elements (Mitchell et al., 2007) such as cognitive style—in terms of intuition and rationality (Chavoushi et al., 2020)—in effective decision making (Nutt, 2008).

Cognitive style refers to an individual’s preferred and habitual approach to organizing, representing, and processing information (Streufert & Nogami, 1989). According to Simon (1972, 1990), people try to be rational, but due to their bounded rationality, they adapt to complex reality by using intuition to aid rather than substitute rational approaches. This explanation implies that rational and intuitive approaches should work together, instead of competing with each other in shaping decision making.

Starting from these theoretical premises, the present study aims to examine the effect of entrepreneurial alertness and the scanning and searching dimension on decision-making effectiveness and the mechanisms of the process. In particular, it tries to investigate how alertness and cognitive style contribute to effective decision-making pathways. It develops and tests a mediation model based on the literature on entrepreneurial cognition and considers intuition and rationality as possible mediators in the relationship between scanning and searching and entrepreneurial decision-making effectiveness.

The model was tested empirically on a sample of 98 entrepreneurs of small and medium manufacturing firms in Tuscany, a region in central Italy, using a survey. The data revealed that the relationship between scanning and searching and decision-making effectiveness was mediated by a rational cognitive style and that both rational and intuitive styles played an important role in effective decision-making processes. The results contribute to the literature by explaining the pathway of thought that leads to effective entrepreneurial decisions and the associations between different cognitive elements (Mitchell et al., 2007; Chauvoushi et al. 2020), namely alertness and cognitive style.

The remainder of the present study is structured as follows. After an explanation of the theoretical background, the conceptual framework and proposed hypotheses are presented. The survey strategy and data architecture are then described. The subsequent section describes the empirical model and methodology. The final sections discuss the findings, and provide an overview of the theoretical and practical implications and suggestions for possible future research.

## Theoretical background: Entrepreneurial cognition, alertness, and decision making

The literature on entrepreneurship has devoted increasing attention to the importance of understanding how entrepreneurs think and the reasons that lead them to do the things they do (Mitchell et al., 2007). Hence, studies of entrepreneurship need to consider the role played by the individual entrepreneur, especially when they have to make decisions (Caputo & Pellegrini, 2019). Significant emphasis has been placed on entrepreneurial cognition. The latter is therefore about understanding how entrepreneurs make use of simplified mental models to connect previously unconnected information that is helpful to generate new ideas, to start a new business, or to cultivate an existing one. Entrepreneurial cognition relies on two key elements (Mitchell et al., 2007): knowledge structures and decision making. Indeed, each entrepreneur is likely to approach decision making in such a way that the differences are related mainly to the content of the entrepreneur’s cognition concerning the decisions they are making (Curseu et al., 2010). This suggests that the way a decision maker’s knowledge structure is composed determines what information will be available during the decision-making process (Coronges et al., 2007).

In the literature on entrepreneurial cognition, a critical role has been assumed by the concept of entrepreneurial alertness (Sassetti et al., 2018). At the root of this concept is Kirzner’s (1979) research. He defines alertness as an individual’s ability to notice opportunities that have been overlooked by others. Over the years, the entrepreneurial alertness literature has evolved, and the cognitive perspective has allowed us to understand this concept more easily (Amato et al., 2017). Certain theories from cognitive science, such as signal detection theory, regulatory focus theory (Baron, 2004), pattern theories (Baron, 2006), schema theories, and attention theories (Valliere, 2013) have assisted in the process. For the purpose of the present study, it is important to underline that entrepreneurial alertness is considered to be the most important cognitive/psychological factor in recognizing entrepreneurial opportunity (Chavoushi et al., 2020), because it is grounded in a distinctive set of perceptual and information-processing skills (Gaglio & Katz, 2001). These skills enable people to organize and interpret information in various domains of knowledge (Gaglio & Katz, 2001), developing cognitive frameworks to build the knowledge structure at the base of entrepreneurial decisions (Mitchell et al., 2002, 2007).

As scholars have recently underlined (Chavoushi et al., 2020), entrepreneurial alertness, as a major cognitive capability, should be investigated using other cognitive approaches such as cognitive style. This is also in keeping with calls to investigate the pathways of thought and the associations between different cognitive elements (Mitchell et al., 2007). Cognitive style refers to an individual’s preferred and habitual approach to organizing, representing, and processing information (Streufert & Nogami, 1989).

Information processing and cognitive style are central to the cognitive psychology literature (Neisser, 1967). Schneider and Shiffrin (1977) identify two kinds of cognitive styles which allow individuals to process information, automatic detection, and undertake controlled searching. Traditionally, this mechanism has been described as a *dual process* that distinguishes between two systems of information processing in the human mind (Chaiken and Trope 1999). On the one side is the non-rational, tacit, and fast system of thought involving the automatic, associative, and selective processing of information (Lieberman, 2000), labelled System 1 (see Kahneman, 2003; Stanovich & West, 2000). On the other side is the rational, deliberate (Hogarth, 2003), and slow system of thought based on the effortful, rule-based, exhaustive processing of information (Sherry & Schacter, 1987). This is termed System 2. Entrepreneurs use two different cognitive processes, rationality and intuition, to process available information (Curşeu et al., 2010; Groves et al., 2011) and to reach a decision. They could therefore be considered as the mediators between knowledge structures, which are built through the scanning and searching dimension of entrepreneurial alertness, and organizational behavior (Davis & Luthans, 1980; Gioia & Manz, 1985; Vermeulen & Curşeu, 2010; Wood & Bandura, 1989). Decision-making effectiveness (Curşeu et al., 2010) is defined as the extent to which decisions achieve the objectives established by the decision makers at the time they are made (Dean & Sharfman, 1996). To examine the effect of entrepreneurial alertness on decision-making effectiveness and the mechanism of this process, the present study develops a mediation model based on the entrepreneurial cognition literature and considers intuition and rationality as mediators of this relationship.

## Hypotheses development

### Entrepreneurial alertness and decision-making effectiveness

There is a consensus in the literature that entrepreneurial alertness consists of three main components: (1) active searching and scanning for opportunities; (2) association and connection; and (3) evaluation and judgment – evaluating the potential value of identified opportunities (Tang, 2009; Tang et al., 2012). Alert scanning and searching refers to constantly scanning the environment and searching for new information, changes, and shifts overlooked by others. Extending alertness as a part of the process of entrepreneurial cognition (Alvarez & Busenitz, 2001; Mitchell et al., 2007), this dimension involves pre-existing knowledge, preparedness, and sensitivity to new opportunities. The second dimension, alert association and connection, involves pulling together disparate pieces of information and building them into coherent alternatives. The third dimension involves making evaluations and judgments about the new changes, shifts, or information and deciding if might present a business opportunity with profit potential. These three components can be considered as schemata that can guide the search for the acquisition, and processing of information, as well as subsequent behavior in response to the gathering of that information (Vallerie, 2013). As recent researchers (Amato et al., 2017) have described, individuals high in entrepreneurial alertness are more likely to form the intention of starting a business (Van Gelderen et al., 2008) and also have greater likelihood than individuals low in alertness to act on these intentions—actually engaging in startup activity (Shane et al., 1991). Furthermore, there is empirical evidence suggesting that alertness is positively related to firm’s innovativeness (Tang et al., 2012). In this context, it is important to note that identifying and selecting the most appropriate and potentially profitable opportunities for a new business are among the most important activities carried out by entrepreneurs (Stevenson et al., 1985).

Previous research has indicated that all three components of entrepreneurial alertness play an important role in a venture’s success (Amato et al., 2017). Nonetheless, the scanning and searching component is fundamental because it is conducted at each stage of the opportunity recognition, development, evaluation, and exploitation process (Tang, 2016). Indeed, as has been noted above, if multiple options survive the screening and searching for new information, they can each be evaluated and the best one chosen (Beach, 1990; Tang, 2016). This is in line with the roots of alertness (Kirzner, 1979, 1985, 1992), which distinguish alert and non-alert individuals with regard to the way they make decisions about their current circumstances. The scanning and search dimension can increase the effectiveness of entrepreneurial decision making. Therefore, we propose the following:

Hypothesis 1: The scanning and search dimension of entrepreneurial alertness has a positive effect on entrepreneurial decision-making effectiveness.

### Entrepreneurial alertness and cognitive style

As Valliere (2013) suggests, the schema theory helps to identify entrepreneurial alertness as a cognitive process. It is one of the most useful and pervasive perspectives on the mechanics of social cognition (Markus & Zajonc, 1985). It argues that schemata guide the search for, the acquisition, and the processing of information, as well as subsequent behavior in response to that information (Harris, 1994). In particular, while they are scanning and searching for information in the environment, alert decision makers accumulate a wide array of domain-relevant information in the form of tacit and explicit knowledge (Polanyi, 1967). They typically have access to far more information than they can actually use (Mintzberg, 1973), and therefore have to be selective in attending to it (Thomas et al., 1993).

Vaghely and Julien (2010) observed that, from an epistemological point of view, decision makers use rational or intuitive models to process the available information acquired from searching and scanning (Tang et al., 2012). Scholars have widely conceptualized rationality and intuition as opposing poles of a single overarching dimension of cognitive style (Allinson & Hayes, 1996). Cognitive style refers to an individual’s preferred and habitual approach to organizing, representing, and processing information (Lord & Maher, 1990; Robey & Taggart, 1981; Streufert & Nogami, 1989). Rational or analytical processing encompasses thinking and decision making that has been variously described as objective, sequential, convergent, logical, and detailed (Sadler-Smith, 2004). For the purpose of the present study, cognitive style is considered as a rational one. Intuitive processing, on the other hand, encompasses thinking and decision making that has been described as divergent, simultaneous, feeling, and holistic. Intuitive processing is therefore considered herein as an intuitive style (Sadler-Smith, 2004).

Entrepreneurs need to look for and assess information to make decisions (Baron, 2006; Shane & Venkataraman, 2000; Short et al., 2010). In this process, the scanning and search dimension of entrepreneurial alertness helps lay the foundation for the development of cognitive frameworks (i.e., prototypes and schemas that reflect an individual’s knowledge and beliefs about the external world). Such cognitive frameworks represent the cumulative experience, learning, and meaning an individual has constructed about a specific domain. They are essential for processing and utilizing stored information and knowledge (Tang et al., 2012). Once this information is stored, it has to be processed. As Vaghely and Julien (2010) explain, during the information-gathering process people try to be rational, but because of their bounded rationality (Simon, 1990) they adapt to the complex reality by using non-rational approaches to aid rather than to substitute rational ones. This implies that rational and intuitive styles are both essential in the elaboration of the information selected by the scanning and search dimension of entrepreneurial alertness (Tang et al., 2012). Based on these theoretical premises, we propose the following:

Hypothesis 2a: The scanning and search dimension of entrepreneurial alertness is positively related to rational cognitive style.

Hypothesis 2b: The scanning and search dimension of entrepreneurial alertness is positively related to intuitive cognitive style.

### Cognitive style and entrepreneurial decision-making effectiveness

An individual’s cognitive style may influence their preference for different types of learning, knowledge gathering, information processing, and decision making (Kickul et al., 2010). The cognitive style is generally thought to have multiple dimensions. These include decision making, learning, personality, and awareness (Leonard et al., 2005). The “awareness” dimension of may be described as a continuum ranging from intuitive to analytic, and has often represented the entire construct of cognitive style (see, e.g., Brigham et al., 2007, who used the label “decision-making style”).

However, subsequent research has found that the intuitive and rational poles are quite distinct (Kickul et al., 2010) and that individuals demonstrate a preference for one over the other (Allinson & Hayes, 1996; Hodgkinson & Sadler-Smith, 2003). This has particular relevance to the entrepreneurship literature. Indeed, entrepreneurs may focus differently on decisions according to their preferred cognitive style, and this may ultimately influence how effective they are. Kahneman (2003) describes two systems that compose the architecture of cognition: intuition (System 1) and reasoning (System 2): “The operations of System 1 are fast, automatic, effortless, associative, and often emotionally charged; they are also governed by habit, and are therefore difficult to control or modify. The operations of System 2 are slower, serial, effortful, and deliberately controlled; they are also relatively flexible and potentially rule-governed” (p. 1451).

There is evidence that cognitive style affects individual strategic decision making in organizations (Gallèn, 2006; Hurst et al., 1989; Leonard et al., 2005). Hurst et al. (1989) found that individuals varied in whether they categorized a problem as strategic or non-strategic. Because preferences in cognitive style continually affect how individuals perceive and interpret information, it is important to understand what impact this has on the entrepreneurial process (Kickul et al., 2010).

Research has demonstrated that the intuitive process is likely to facilitate the entrepreneur’s decision-making process (Dane & Pratt, 2007; Groves et al., 2011; Sadler-Smith & Shefy, 2004). Rational thinking, however, may also be useful, because it can reduce the negative consequences and cognitive biases that may arise (Groves et al., 2011; Simon et al., 2000). Hence, as Fiet (2002) demonstrates, rational thinking is a crucial element in entrepreneurial success during the opportunity discovery process. It is important to underline that venture success can be considered as a measure of effectiveness (Nutt, 2008), in particular decision-making effectiveness. Groves et al. (2011) argue that “…in contrast to a popular nonlinear, stereotype of entrepreneurs as being primarily creative, visionary, and intuitive,.. entrepreneurs utilize both nonlinear and linear dimensions in their overall cognitive processes, and employ either a linear or a nonlinear thinking style depending on situational circumstances and the different entrepreneurial and functional needs within an enterprise” (p. 444). The authors stress that entrepreneurs are much more balanced and versatile in their use of rational and intuitive styles than their professional counterparts (Groves et al., 2011). In light of these premises, we propose the following:

Hypothesis 3a: The use of rational style has a positive effect on entrepreneurial decision-making effectiveness.

Hypothesis 3b: The use of intuitive style has a positive effect on entrepreneurial decision-making effectiveness.

### The mediation effect of cognitive style between entrepreneurial alertness and decision-making effectiveness

Information “ alters mental representation […], reduces uncertainty and rich information helps make sense of ambiguous situations” (Vaghely & Julien, 2010, p. 75). Alertness, in particular the scanning and search dimension, enables persons to organize and interpret information in various domains of knowledge related to the development of new opportunities (Gaglio & Katz, 2001). As has been noted, the scanning and search dimension helps lay the foundation for developing cognitive frameworks to build the knowledge structure at the base of the entrepreneurial decision-making process (Mitchell et al., 2002, 2007). Entrepreneurs who scan and search more extensively will have a wider range of information (Gaglio, 1997), which can make them more alert to business opportunities (Ericsson, 1999). Once that information becomes part of the entrepreneurs’ knowledge structure, it has to be processed through their particular cognitive style (Streufert & Nogami, 1989). Because they may use two different cognitive processes (Groves et al., 2011; Curşeu et al., 2010) to reach a decision, they could be considered as mediators between the knowledge structure that is built through the scanning and search dimension of entrepreneurial alertness and organizational behavior (Davis & Luthans, 1980; Gioia & Manz, 1985; Vermeulen & Curşeu, 2010; Wood & Bandura, 1989), specifically decision-making effectiveness (Curşeu et al., 2010). Based on these theoretical premises, we propose the following:

Hypothesis 4a: Rational style mediates the relationship between the scanning and search alertness dimension and entrepreneurial decision-making effectiveness.

Hypothesis 4b: Intuitive style mediates the relationship between the scanning and search alertness’ dimension and entrepreneurial decision-making effectiveness.

**Fig. 1:**
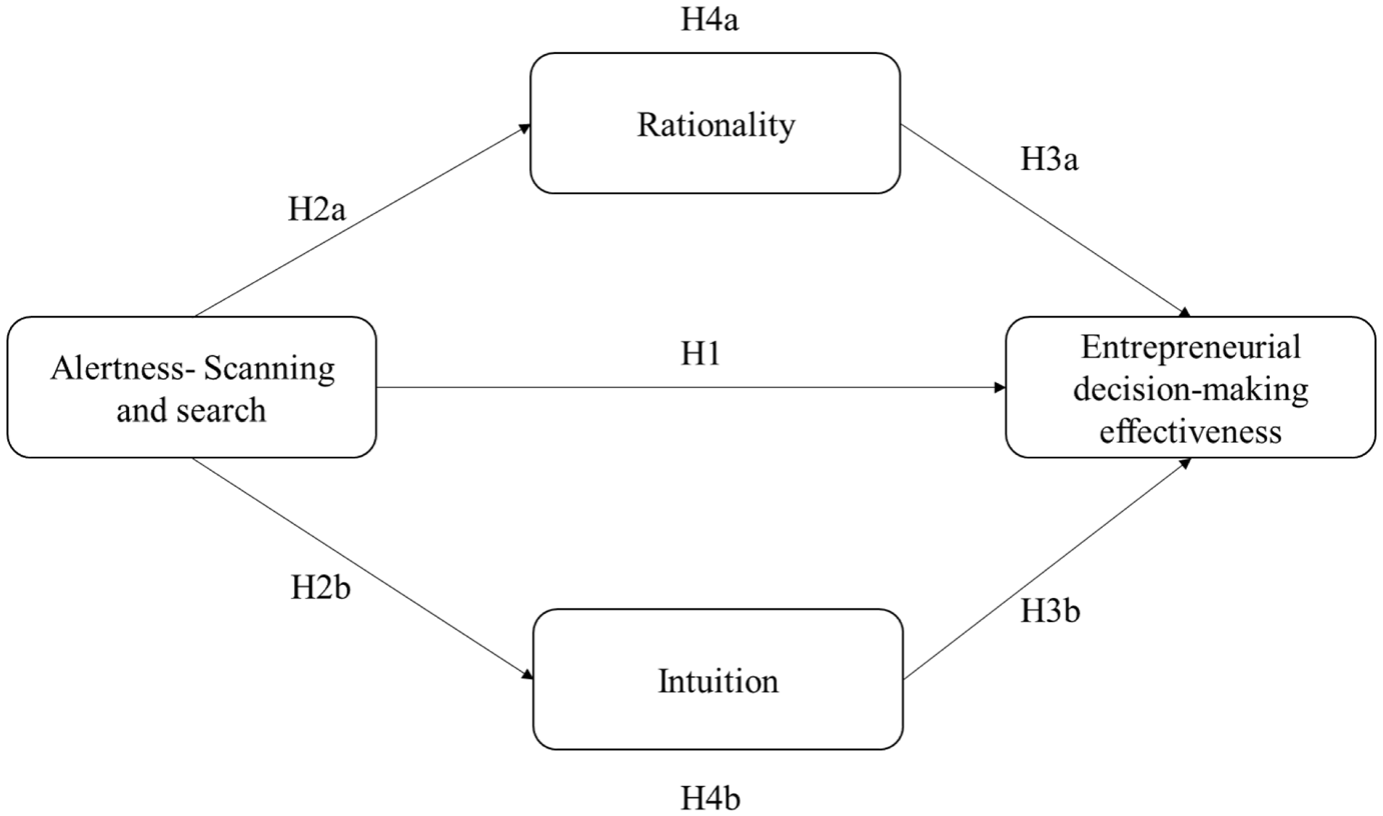
Theoretical framework

The hypotheses are presented in Fig. 1.

## Method

### Sample and data collection

Data were collected through a questionnaire distributed among a sample of entrepreneurs of small and medium manufacturing firms in Tuscany, central Italy. According to the Regional Institute for Tuscany’s Economic Program (IRPET, 2016), the Tuscan economy is based mostly on manufacturing activities, in which 13% of companies are involved. The fashion and mechanical industries are among the most important. The fashion segment (which includes textiles, clothing, and leather) accounts for 5.3% of the region’s business, compared with 2% for the national economy. The mechanical segment (which includes leather goods and turbines) is another leading economic driver of the regional economy, with plant located in different cities. As IRPET reports, these segments are also key exporters.

To identify an appropriate sample of companies for the present study, the European Commission criteria for small and medium-sized enterprises (SMEs) were considered: firm size (between 10 and 250 employees) and turnover (below €50 million). The research population was composed of 3,734 SMEs listed at the Chamber of Commerce^1^ of Florence; 2,320 belonged to the fashion industry and 1,414 to the mechanical industry. In keeping with the literature (Elzahar et al., 2015), and to make our sample as representative as possible, we used a systematic sampling approach. As researchers have acknowledged, this technique is operationally more convenient than simple random sampling. Hence, each unit had an equal probability of inclusion in the sample. The first unit was selected with the help of random numbers, and the remaining ones were selected automatically according to a predetermined pattern. As a result, the systematic sample was spread more evenly over the population.

To apply systematic sampling, the firms included in the overall population were randomly selected so that each industry, provincial area, and number of employees were represented in the same proportion as in the initial sample. For this purpose, all of the firms were ranked in each industry by numbers of employees and provincial area. After this, we retained the first firm in every industry as a starting point, then the third, the fifth, and so on. Eventually, the total amount of firms that took part in the study was 1,397. Given the sampling technique adopted, we were confident that our sample was strongly representative of the entire population of Tuscan SMEs.

To administer the questionnaire, an online search for company email addresses and telephone number was carried out; in case any were not available, the initial database was used to consider alternative firms, while respecting the number of employees, the type of industry, and the provincial area. Some observations were excluded due to data unavailability. The final sample consisted of 1,261 SME entrepreneurs (755 fashion firms and to 506 mechanical companies). Data were collected via a web-based questionnaire following the procedure suggested by Dillman et al. (2014); 1,261 emails were sent to the entrepreneurs included in the sample. After the first invitation, which was sent in March 2017, 23 questionnaires were returned: 11 were completed, and 12 were not. Three rounds of reminders, one per week, were sent out, which generated another 87 complete responses. The final sample comprised 98 entrepreneurs.

Among these, 77.55% were male and 22.45% female. The sample comprised 60.20% fashion companies and 39.80% mechanical ones; the average business tenure was 34.93 years; 82.65% were small enterprises (10 − 49 employees) and 17.35% medium (50 − 250 employees). The average sample size was rather small, but this was similar to other studies that have considered entrepreneurs’ cognitive variables (Baron & Tang, 2011; Baron et al., 2011; Brigham et al., 2007).

To check the possible confounding effects resulting from nonresponse bias, differences between early and late respondents were examined (Fowler, 1993). A number of *t-*tests were performed, comparing these sub-groups in terms of demographic and model variables. Given that no significant differences were found, the conclusion was that the dataset did not suffer from such bias.

### Measurement scales

The variables were operationalized through self-perception measures (Spector, 1994) based on a 5-point Likert-type scale, from 1 (*strongly disagree*) to 5 (*strongly agree*). Existing research (Oh et al., 2011) has shown that self-reporting enables researchers to measure variables that are not verifiable by other means, because it can capture personality traits, individuals’ intentions, attitudes, and orientations that are, by nature, inherent to the respondent. Previous studies on alertness (e.g., Amato et al., 2017; Tang, 2016; Tang et al., 2012) and decision making (Baron & Tang, 2011; Baron et al., 2011; Groves et al., 2011) have consistently used self-perception measures.

*Independent variable: Entrepreneurial alertness scanning and searching:* Following recent studies (e.g., Zhao et al., 2020), entrepreneurial alertness was measured using Tang et al.’s (2012) 6-item scale. The Cronbach’s alpha coefficient was 0.85.

*Mediators: Cognitive style.* The intuitive style was measured using a combination of items taken from different studies. The first two items belonged to the decision-making style scale from Covin et al. (2001), while the final three were part of the intuitive synthesis scale used in Khatri and Ng (2000)’s study. The merger of these items allowed us to capture the idea that intuition is rooted in experience, and superior intuition is often developed through exposure to varied experiences in which relationships between phenomena can be more fully understood (Khatri & Ng, 2000). We adopted a scale that focused more on intuition as a product of experience rather than emotion. The Cronbach’s alpha coefficient was 0.81. Betsch and Kunz’s (2008) 6-item scale was used to measure rational style. The respondents were asked to evaluate the way they generally make innovation/internationalization decisions on a scale with anchors 1 = *totally disagree* to 5 = *strongly agree*. Cronbach’s alpha was 0.90.

*Dependent variable: Decision-making effectiveness.* Based on Jansen et al. (2013)*,* four items were used to measure the dependent variable. They formed a 3-point Likert scale (from 1 = *a few* to 3 = *a great deal*). Respondents were asked to what extent their innovation/internationalization decisions had contributed to (1) turnover and (2) profits; (3) how far they were satisfied with their decisions; and (4) whether those decisions had led to the expected result. Decision effectiveness was calculated as the sum of these items.

Table 1 provides a summary of the measurement scales adopted, including the item-to-total correlation which, in all cases, was above the downward threshold of 0.50 recommended in the literature (Tian et al., 2001).

**Table 1:** Measurement Scales, Items, Cronbach’s Alpha and Item-to-Total Correlations for All Variables

Variable	Items	Source	Item sample	Cronbach’s alpha	Item-to-total correlations
Scanning and searching	6	Tang et al. (2012)	“I always keep an eye out for new business ideas when looking for information”	0.85	SCAN1: 0.64
					SCAN2: 0.68
					SCAN3: 0.55
					SCAN4: 0.51
					SCAN5: 0.71
					SCAN6: 0.75
Rationality	6	Betsch and Kunz (2008)	“Before making decisions, I first think them through”	0.90	RATION1: 0.83
					RATION2: 0.71
					RATION3: 0.70
					RATION4: 0.61
					RATION5: 0.75
					RATION6: 0.82
Intuition	5	Covin et al. (2001); Khatri and Ng (2000)	“On many occasions, senior managers do not have enough information, and must make important decisions based on gut feeling”	0.81	INTUIT1: 0.72
					INTUIT2: 0.59
					INTUIT3: 0.48
					INTUIT4: 0.63
					INTUIT5: 0.59
Decision- making effectiveness	4	Jansen et al. (2013)	“To what extent did the strategic decision contributed to turnover growth?”	–-	–-

### Common method variance

Because a well-known issue with self-reported measures is common method variance (CMV), the present study followed an *ex-ante* research design strategy in addition to performing *ex-post* tests. In the case of the former, we ensured that the questionnaire was wide ranging enough and that it included constructs beyond those investigated in the present study. The scales measuring the mediators and the independent and dependent variables were separated and the survey as a whole was designed in such a way as to guarantee the respondents’ anonymity. The formulation of the items was checked to ensure that the questions were understandable. Podsakoff et al. (2003) point out that these procedures tend to be highly useful in that they “reduce people’s evaluation apprehension and make them less likely to edit their responses to be more socially desirable, lenient, acquiescent and consistent with how the researcher wants them to respond” (p. 888). With regard to the *ex-post* tests, Harman’s (1967) one-factor test was performed using principal component factoring. If a relevant amount of CMV is present, analysis can result in either a single factor or one general factor accounting for most of the covariance in the independent and dependent variables (Lings & Greenley, 2005). After performing both unrotated and rotated factor analyses using the eigenvalue greater-than-one criterion, three factors were revealed; the first accounted for 35% of the variance, which was below the threshold value of 50% recommended by Podsakoff et al. (2003). Taken together, the findings therefore suggested that CMV was not an issue.

### Confirmatory factor analysis and scales’ reliability

Before testing the proposed hypotheses, confirmatory factor analysis (CFA) was run using LISREL 8.80 to check the adequacy of the measures adopted (see Table 2). All items loaded well on their predicted factor. The CFA findings demonstrated that the model fitted the data well. Because of the importance of providing more than one index (e.g., Černe et al., 2013), the IFI (Incremental Fit Index = 0.98), TLI (Tucker Lewis Index/Non-Normed Fit Index = 0.98), CFI (Comparative Fit Index = 0.98), and RMSEA (= 0.04) were calculated, and all showed a good fit (Hu & Bentler, 1999). The measurement model also supported convergent validity. Hence, the average variance extracted (AVE) of most of the constructs was greater than 0.50. Only the intuition construct showed a slightly lower value (i.e., 0.47). Nonetheless, when all composite reliability (CR) estimates are above 0.70 (Hair et al., 2010), scholars acknowledge that the convergent validity of the constructs can still be considered adequate (see Fornell & Larcker, 1981). In addition, discriminant validity was checked by comparing the AVE for a given construct with the maximum value among the square roots of the correlations between that construct and others. The results showed that all constructs had an AVE higher than the corresponding maximum squared inter-construct correlation (Hair et al., 2010; Henseler et al., 2015). Overall, the results supported the validity of the measures used.

**Table 2:** Confirmatory Factor Analysis Results and Construct Validity

Construct	Item	Unstandardized Coefficient Estimate (SE)	Standardized Coefficient Estimate*	AVE	CR
Scanning and Search (SCAN)	SCAN1	1.000	0.716	0.51	0.86
	SCAN2	1.153 (0.153)	0.813		
	SCAN3	0.916 (0.177)	0.554		
	SCAN4	0.890 (0.187)	0.512		
	SCAN5	1.113 (0.158)	0.760		
	SCAN6	1.289 (0.163)	0.858		
Rationality (RATION)	RATION1	1.000	0.875	0.62	0.91
	RATION2	0.787 (0.085)	0.769		
	RATION3	0.795 (0.090)	0.743		
	RATION4	0.792 (0.111)	0.641		
	RATION5	0.837 (0.083)	0.809		
	RATION6	1.019 (0.089)	0.869		
Intuition (INTUIT)	INTUIT1	1.000	0.822	0.47	0.82
	INTUIT2	0.842 (0.129)	0.683		
	INTUIT3	0.657 (0.128)	–0.544		
	INTUIT4	0.977 (0.141)	–0.727		
	INTUIT5	0.878 (0.144)	0.642		
Decision making effectiveness (DMEFF)	–-	1.000	–-	–-	–-

*p < 0.001. NC [X^2^/df] = 1.26, *p* < 0.001, IFI = 0.98, TLI (NNFI) = 0.98, CFI = 0.98, and RMSEA = 0.04

## Results

Descriptive statistics and correlation coefficients for all variables are reported in Table 3. The variables observed showed no coefficient above 0.80, a threshold value indicating the risk of multicollinearity in the data (Kennedy, 1985).

**Table 3:** Descriptive Statistics and Correlation Matrix (n = 98)

Construct	Mean	St. Dev	Min	Max	1	2	3	4
1. Scanning and searching	3.70	0.77	1	5	*-*			
2. Rationality	4.05	0.74	1	5	0.65^***^	*-*		
3. Intuition	2.65	0.82	1	5	0.06	–0.06	*-*	
4. Decision-making effectiveness	8.88	1.90	4	12	0.27^***^	0.36^***^	0.18	*-*

*** Correlation is significant at the 0.001 level

In accordance with recent research (see González-Serrano et al., 2020; Huezo-Ponce et al., 2020; Rwehumbiza & Marinov, 2020), to test the hypotheses, SPSS (v.26) was employed as a tool for the regression data analysis. As Table 4 shows, the scanning and search alertness dimension was a significant predictor of entrepreneurial decision-making effectiveness (β = 0.631, *p* = 0.01), so Hypothesis 1 was confirmed. The results of the statistical analysis also revealed a strong support for the relationship between the scanning and search alertness dimension and rationality (Hypothesis 2a, β = 0.569, *p* < 0.000). The relationship between alertness scanning and search and intuition was not significant, so Hypothesis 2b was not supported. Further, in line with Hypotheses 3a and 3b, the results demonstrated that both rationality (β = 0.941, *p* < 0.000) and intuition (β = 0.490, *p* < 0.05) had a significative and positive effect on entrepreneurial decision making, but that the rationality effect was stronger than intuition.

**Table 4:** Regression Test Results

Path/effect	Standardised		
	β	SE	*p*
Scanning and searching → Decision-making effectiveness	0,6310	0,2550	0,0110
Scanning and searching → Rationality	0,5690	0,0790	0,0000
Scanning and searching → Intuition	–0,0550	–0,0520	0,6140
Rationality → Decision-making effectiveness	0,9410	0,2420	0,0000
Intuition → Decision-making effectiveness	0,4900	0,2180	0,0270

Finally, it was expected that cognitive styles, namely rationality and intuition, would mediate the relationship between the alertness scanning and search dimension and entrepreneurial decision-making effectiveness (Hypotheses 4a and 4b). To test this, and in line with recent studies (see, e.g., Linder & Nippa, 2019; Zhao et al., 2020) the SPSS-Process Macro Plugin (Hayes, 2013) was used, which is based on the regression-based bootstrapping approach. This method provides an effective tool to obtain confidence limits for indirect effects, and is the most highly recommended method to test for mediation. It relies on resampling the data, whereby one draws a large number (e.g., 5,000) of new samples of size (n) from the original sample (Lombardi et al., 2019). The model parameters are estimated for each new sample, resulting in many estimates for each parameter. PROCESS mediation “directly tests the indirect effect between the predictor and the criterion variables through the mediator via a bootstrapping procedure, addressing some weaknesses associated with the Sobel test” (Huertas-Valdivia et al., 2018, p. 227).

The present study adopted the theoretical model 4 with two mediators. As Table 5 shows, the results (which were based on 5,000 bootstrap examples) indicated that the 95% confidence intervals for the indirect effect of rational style between the alertness scanning and search dimension and entrepreneurial decision-making effectiveness did not contain zero, validating Hypothesis 4a (confidence interval 95% [0.0176; 1.0070]). Hypothesis 4b was not supported (confidence interval 95% [-0.1541, 0.1447]) so intuitive style was not considered a mediator between the scanning and search dimension of alertness and entrepreneurial decision-making effectiveness.

**Table 5:** Mediation Test Results

Indirect effect		95% confidence interval*	
	Boot SE	Boot LLCI	Boot ULCI
Scanning and search—> rationality—> Decision-making effectiveness	0,1954	0,1104	0,8787
Scanning and searching—> intuition—> Decision-making effectiveness	0,0582	–0,1605	0,0796

*5,000 bootstrap samples for bias-corrected bootstrap confidence intervals

## Discussion

### Theoretical contributions

Inspired by the entrepreneurial cognition literature (Mitchell et al., 2007), the present study set out to examine the impact of entrepreneurial alertness on decision-making effectiveness and the role played by cognitive style, both intuitive and rational, in this relationship. It has explained why some alert entrepreneurs make effective decisions and others do not. Broadly speaking, the study makes four important contributions to the existing research on this topic.

First, it has examined the relationship between the scanning and search dimension of entrepreneurial alertness and decision-making effectiveness. Previous studies have demonstrated that individuals high in entrepreneurial alertness are more likely to form the intention of starting a business (Amato et al., 2017; Van Gelderen et al., 2008). They also have a greater likelihood than individuals low in alertness to act on these intentions, for example by engaging in startup activity (Shane et al., 1991). Furthermore, there is empirical evidence suggesting that alertness is positively related to a firm’s innovativeness (Tang et al., 2012). Nevertheless, the precise effect of the scanning and search component of alertness, which is considered to be the main pillar of this process because it is conducted at each stage of the opportunity recognition, development, evaluation, and exploitation process (Tang, 2016), has not been closely examined. The present study has enriched this discussion by investigating the consequences of entrepreneurial alertness (Chavoushi et al., 2020). It has demonstrated the importance for entrepreneurs to apply their competence to scan and search information not only to recognize, develop and evaluate business opportunities (Tang, 2016), but also to exploit them through effective decisions making.

Second, the results revealed that the relationship between the scanning and search dimension of entrepreneurial alertness and decision-making effectiveness was mediated by a rational cognitive style. This explains the pathway of thought and associations between different cognitive elements (Chauvoushi et al., 2020; Mitchell et al., 2007), namely, alertness and cognitive style. In particular, the study has demonstrated that the scanning and search dimension of entrepreneurial alertness is fundamental in developing and storing cognitive frameworks and information (Baron, 2006; Shane & Venkataraman, 2000; Tang et al., 2012) that have to be processed by rationality before effective entrepreneurial decisions are made (Vaghely & Julien, 2010). This is in keeping with Tang et al. (2012) proposition: that the basic rationale for the systematic search view is that opportunity identification depends on a fit between entrepreneurs’ prior knowledge and a particular venture idea, which may be discovered via systematic searching (Fiet, 2002). Tang et al. (2012) does not confirm this hypothesis, and the present study explains why. To make effective decisions, entrepreneurs have to be systematic and rational when processing and interpreting prior knowledge and information.

Third, most previous studies have investigated separately the effect of rationality (Fiet, 2002) and intuition (Jansen et al., 2013) on entrepreneurial decision-making effectiveness. Few contributions have considered the combined effect of intuition and rationality on entrepreneurial venture performance. Hypothesizing that both intuition and rationality may have an impact on entrepreneurial performance, Sadler-Smith (2004) found that only intuition had a positive effect on financial and non-financial results. The present study brings clarity to the suggestions that both rationality and intuition are critically important in the effectiveness of entrepreneurial decision making (Groves et al., 2011). It has demonstrated that, while both intuition and rationality are important in the effectiveness of the decision-making process, rationality has a greater impact than intuition on the effectiveness of entrepreneurial decisions.

Finally, the present study contributes to the literature by showing how entrepreneurs make use of simplified mental models to connect previously unrelated information that might be helpful in generating new ideas, starting a new business, or fostering an existing one, by revealing the relationship between the two main elements of entrepreneurial cognition: knowledge structures and decision making (Mitchell et al., 2007). In particular, the findings confirm that entrepreneurs’ knowledge structures can be constructed through the scanning and search dimension of entrepreneurial alertness (Davis & Luthans, 1980; Gioia & Manz, 1985; Vermeulen & Curşeu, 2010; Wood & Bandura, 1989) and that decision-making effectiveness can be attained through a rational decision-making process.

### Practical implications

The findings have a number of implications for entrepreneurs and the management of their ventures. In particular, they suggest that to make effective decisions, entrepreneurs not only need to develop the competence to be alert to their environment, searching and scanning specific information, but also to develop rational ways of thinking. In other words, while it is essential for them to be more alert to fundamental and critical information, they must also be able to elaborate on this information, to transform the *alert moment* into the *decision moment* that will determine the effectiveness of their decision making. The results of the study underline the fact that the accumulation of information from the environment through alert scanning and searching is not enough if it is not then processed through the application of a rational cognitive style. Their ventures will survive and thrive when the input is translated into effective decision making.

Consequently, the present study helps entrepreneurs to understand the way they make decisions. Entrepreneurial awareness is defined as the propensity to notice and to be sensitive to information about their behaviour in the environment, and to discover new needs and opportunities (Ardichvili et al., 2003). Therefore, those who succeed in doing so have the chance to make more effective decisions and ultimately to perform more successfully (Sassetti et al., 2019). In general, entrepreneurs have to be aware that entrepreneurial decision making is a complex and multifaceted process in which different cognitive aspects are linked to each other (i.e., alertness and cognitive style); an understanding of these processes will enrich their knowledge of the mechanisms that motivate them to participate in the economy, thereby generating growth and prosperity (Vermeulen & Curseu, 2010).

The present study has revealed that entrepreneurs are aware that the effectiveness of their decisions depends on an interplay between the alertness scanning and search dimension and a rational cognitive style. In particular, the decision-making process should start with the scanning and searching of information, enabling entrepreneurs to organize and interpret what they discover in the various domains of knowledge related to the development of new opportunities (Gaglio & Katz, 2001); once the information become part of the entrepreneurs’ knowledge structure, it then has to be processed. The entrepreneurs studied understood that when strategic decisions have to be made, the simple activation of pre-existing knowledge structures from long term memory is not sufficient (Vermeulen & Curseu, 2010) and that intuitive information processing (System 1) alone is not effective. Therefore, the information that is available needs to be evaluated carefully, new information needs to be gathered, and eventually new task-specific knowledge representations need to be created; a more rational and controlled information processing (System 2) should therefore also be applied.

The results of the present study also have implications for entrepreneurship education. Alertness is considered to be a distinctive set of perceptual and information-processing skills (Gaglio & Katz, 2001). Given this, it can likely be taught and developed through entrepreneurship programs. It would be useful to build experiential learning, case studies, or business simulations that allow established and future entrepreneurs to develop their ability to scan and search information within their competitive environment. Moreover, these methods would be useful in developing critical and more rational thinking and in explaining and underlining the importance of detecting information in the environment and elaborating on it in a clear and rational way so as to make effective decisions. Entrepreneurship programs typically emphasize the importance of seizing business opportunities through intuition, creativity, and out of the box thinking. It is undeniable that they are important—and the results of the present study support this view—but they should also be used to train future entrepreneurs to think in more deliberative ways.

### Limitations and future research directions

The present study has a number of limitations. First, although it began from the premise that the scanning and searching dimension of entrepreneurial alertness is fundamental for business success (Amato et al., 2017), it did not take into consideration the other two dimensions of alertness: association and connection and evaluation and judgment (Tang et al., 2012). For this reason, future researchers might investigate the relationship between these dimensions and entrepreneurial decision-making effectiveness and verify if these connections are explained by cognitive styles. Moreover, it would be interesting to test the relationships between the three alertness components (Tang et al., 2012) and their influence on entrepreneurial decision-making effectiveness and to verify whether one had a dominant effect over the others.

In answering to the call for an understanding of the pathways of thought and the association between alertness and other cognitive elements (Chavoushi et al., 2020; Mitchell et al., 2007), the present study focused on cognitive style (Mitchell et al., 2007). Future researchers could apply other cognitive theories such as metacognition, social cognition, and counterfactual thinking (Chavoushi et al., 2020). The cognitive lens would be helpful in beginning to grasp the implications of COVID-19 for entrepreneurship (Giones et al., 2020; Kuckertz et al., 2020). Indeed, to move forward, it will not be enough to wait for things to go back “to normal” (Giones et al., 2020). It is necessary to build an alert mindset to be ready for new entrepreneurial opportunities that may emerge from these turbulent times.

Third, the results showed no significant relation between the scanning and search dimension of alertness and intuitive style. This may be because the focus was on one of the three dimensions of entrepreneurial alertness. Future researchers could therefore examine whether the other two alertness components, association and connection and evaluation and judgment, have an association with intuitive style, and whether intuition has a mediating effect on the dimensions of alertness and decision-making effectiveness. Previous studies have demonstrated that evaluation and judgment have a significant relationship with intuition (Tang et al., 2012), but other cognitive mechanisms could still be investigated.

Finally, in accordance with recent research (i.e., Linder & Nippa, 2019; Zhao et al., 2020), the present study used a cross-sectional design for the questionnaire, which naturally made it difficult to assess causality relationships. In the future, longitudinal or secondary data could be collected to test the hypotheses. Further qualitative analysis might also help in this regard; researchers could carry out interviews, case studies, and text analysis to understand more fully the interplay between alertness, cognitive style, and decision-making effectiveness.

## Footnotes

1 The Chamber of Commerce is a form of local business network whose purpose is to support the interests of businesses, contributing to local development. Previous researchers have noted the important role played by these organizations: “the chambers appear on the surface to be powerful organizations with a strong potential for local economic growth. Indeed, they have greater formal powers and provide a wider representation of interests than any other organization at local level besides the local authorities themselves. Chambers are of special interest in that they seem to embody all the attributes of a ‘cooperative endogenous network’” (Waters, 1999, p. 116).
